# Differences in Biofilm Formation of *Listeria monocytogenes* and Their Effects on Virulence and Drug Resistance of Different Strains

**DOI:** 10.3390/foods13071076

**Published:** 2024-04-01

**Authors:** Yujuan Yang, Xiangxiang Kong, Bing Niu, Jielin Yang, Qin Chen

**Affiliations:** 1 School of Life Sciences, Shanghai University, Shanghai 200444, China; yangyj@shu.edu.cn (Y.Y.); bingniu@shu.edu.cn (B.N.); 2 Institute of Translational Medicine, Shanghai University, Shanghai 200444, China; kongxiangxiang@shu.edu.cn; 3 School of Medicine, Shanghai University, Shanghai 200444, China; 4 Technical Centre for Animal, Plant and Food Inspection and Quarantine of Shanghai Customs, Shanghai 200135, China

**Keywords:** extracellular polymeric substances, invasion, adhesion, antibiotic

## Abstract

*Listeria monocytogenes* is recognized as one of the primary pathogens responsible for foodborne illnesses. The ability of *L. monocytogenes* to form biofilms notably increases its resistance to antibiotics such as ampicillin and tetracycline, making it exceedingly difficult to eradicate. Residual bacteria within the processing environment can contaminate food products, thereby posing a significant risk to public health. In this study, we used crystal violet staining to assess the biofilm-forming capacity of seven *L. monocytogenes* strains and identified ATCC 19112 as the strain with the most potent biofilm-forming. Subsequent fluorescence microscopy observations revealed that the biofilm-forming capacity was markedly enhanced after two days of culture. Then, we investigated into the factors contributing to biofilm formation and demonstrated that strains with more robust extracellular polymer secretion and self-agglutination capabilities exhibited a more pronounced ability to form biofilms. No significant correlation was found between surface hydrophobicity and biofilm formation capability. In addition, we found that after biofilm formation, the adhesion and invasion of cells were enhanced and drug resistance increased. Therefore, we hypothesized that the formation of biofilm makes *L. monocytogenes* more virulent and more difficult to remove by antibiotics. Lastly, utilizing RT-PCR, we detected the expression levels of genes associated with biofilm formation, including those involved in quorum sensing (QS), flagellar synthesis, and extracellular polymer production. These genes were significantly upregulated after biofilm formation. These findings underscore the critical relationship between extracellular polymers, self-agglutination abilities, and biofilm formation. In conclusion, the establishment of biofilms not only enhances *L. monocytogenes*’ capacity for cell invasion and adhesion but also significantly increases its resistance to drugs, presenting a substantial threat to food safety.

## 1. Introduction

*Listeria monocytogenes* (*L. monocytogenes*) is a Gram-positive, rod-shaped bacterium with zoonotic capabilities, known for causing listeriosis upon infection [1]. This pathogen demonstrates remarkable tolerance to external conditions, including high salt concentrations, acidic, and alkaline conditions [2]. It can proliferate within a temperature range of 0–45 °C and maintain viability under refrigeration for extended periods, earning it the moniker “refrigerator killer” due to its potential threat to human health [3]. Recognized by the World Health Organization (WHO) as one of the four major foodborne pathogens, *L. monocytogenes* is subject to stringent regulations in China, where its presence in food is strictly prohibited by national food safety standards. In humans, infection with *L. monocytogenes* can lead to severe clinical outcomes, including bacteremia, monocytopenia, meningitis, and so on [4,5]. This bacterium can break through the body’s protective barrier, resulting in a mortality rate of between 20 and 40% in infected people. Notably, *L. monocytogenes* tends to form biofilms, allowing it to be planted on the surface of food industry-related equipment made from stainless steel, high-density polyethylene materials, and glass [6]. This greatly increases the probability of food contamination. Biofilm formation enhances *L. monocytogenes*’ resilience against adverse conditions such as dryness and antibiotics, rendering complete eradication challenging [7]. The formation of biofilm also upregulates its efflux pump MdrL, thereby enhancing the tolerance of *L. monocytogenes* to benzalkonium chloride (BC) and greatly increasing its viability in food [8].

Bacterial biofilms are structured communities of cells that adhere to biological or non-biological surfaces during their growth process. The primary structural components of bacterial biofilms include extracellular polysaccharides, proteins, and DNA [9]. This formation is a strategic adaptation by bacteria to enhance their survival in natural environments [10]. *L. monocytogenes* often exists in the form of biofilms, and biofilm formation is a process regulated by a variety of factors. The formation process of biofilms is the transition from plankton to the state of envelope [11], and there are multi-component changes in the formation process. Surface hydrophobicity and self-agglutination ability helped bacteria enhance adhesion and cell-to-cell recognition. According to Borghi et al. [12], cell surface hydrophobicity is an important predictor of *Candida* biofilm formation. Heo et al. showed that the PTS (phosphotransferase system) component EIIAGlc was able to regulate the intracellular concentration of c-di-GMP, thereby regulating biofilm formation [13]. In addition, quorum sensing systems (QS) and two-component systems (TCS) also play a regulatory role in the formation of biofilms. In *L. monocytogenes*, *luxS*-deficient strains exhibites stronger biofilm-forming ability, but exogenous addition of the AI-2 precursor S-ribosylhomocysteine (SRH) can restore the biofilm-forming ability of wild strains [14]. In TCS, *degU* has been confirmed to play an important role in biofilm formation in *L. monocytogenes*. *degU* is thought to lead to increased biofilm formation by altering cell surface structure or some unknown biochemical pathways [15]. In addition, the global regulator *SpoVG* [16], ABC transporter *VirAB* [17], and *hfq* [18] gene all play a crucial role in the formation of biofilms. Research indicates that pathogenic bacteria within biofilms are challenging to eradicate completely, contributing to persistent food contamination issues. Most of these pathogenic bacteria exist in biofilm states, with over 60% of human bacterial infections being associated with biofilms. The regulatory mechanism of bacterial biofilms plays an extremely important role in the formation, maintenance, and function of biofilms [19]. Consequently, comprehending these regulatory mechanisms offers a novel strategy for inhibiting the formation of *L. monocytogenes* biofilms, presenting new avenues for enhancing food safety and public health [20].

Previous studies have only examined the influence of individual factors on the biofilm formation ability of *L. monocytogenes*, without considering the differences in biofilm formation abilities among strains. Additionally, they have only observed the differences in antibiotic resistance before and after biofilm formation with relatively limited antibiotics. Building on this foundation, our study investigated the differences in biofilm formation ability among different standard strains of *L. monocytogenes* and demonstrated the relationship between extracellular polymers, surface hydrophobicity, auto aggregation capability, and biofilm formation ability. Furthermore, we conducted antibiotic resistance experiments using 14 antibiotics to comprehensively understand the antibiotic resistance situation before and after biofilm formation in *L. monocytogenes* and discussed the changes in its cell invasion and adhesion capabilities during biofilm formation. Overall, this study aims to deepen our understanding of the mechanisms of biofilm development and its impact on the pathogenicity and antibiotic resistance of *L. monocytogenes*.

## 2. Materials and Methods

### 2.1. Bacterial Strains and Activation

*L. monocytogenes* obtained from the Technical Centre for Animal, Plant, and Food Inspection and Quarantine of Shanghai Customs were retrieved from glycerol cryopreserved solution. The thawed bacterial solution was streaked and cultured on tryptone soybean agar plates (TSA-YE, Beijing Bridge Technology Co., Ltd., Beijing, China). Single colonies were picked and cultured in soy broth with 0.6% yeast extract (TSB-YE, Beijing Bridge Technology Co., Ltd.) medium overnight at 37 °C at 200 rpm (Shaker, Being, Ontario, CA, USA).

### 2.2. Detection of the Biofilm-Forming Capacity of L. monocytogenes

Take the bacterial solution cultured overnight, and then dilute this overnight culture to an optical density (OD) at 600 nm of 0.8 (equivalent to approximately 10^8^ CFU/mL) using a microplate reader (Molecular Devices) for subsequent experiments. The prepared bacterial solution was mixed at a ratio of 1:100 and subsequently transferred to a fresh TSB-YE medium. Then, 200 mL of mixed bacterial solution was added to a sterile 96-well plate, sealed with parafilm, and incubated at 37 °C for 1–4 days (Oven, Kenton, Guangzhou, China). TSB-YE medium without any bacterial solution was used as a blank control.

The assessment of biofilm formation using crystal violet staining was conducted following the methodology described by Crespo et al., with further enhancements made to refine the process [21]. The cultured biofilm supernatant was removed, and each well was washed twice with 200 μL of double-distilled water to remove non-adherent bacteria from the bottom of the well. Then, wells were dried at 55 °C. Subsequently, 200 μL of 0.1% crystal violet staining solution was added to each well and stained at room temperature for 45 min. The staining solution was absorbed. Then, the plate was washed twice with double-distilled water to completely wash off the unreacted staining solution, and dried again at 55 °C. After air-drying, 200 μL of 95% ethanol (Sinopharm Chemical Reagent Co., Ltd., Shanghai, China) was added to each well for destaining. After 30 min of destaining, the absorbance at OD_595_ was measured.

### 2.3. Observation of Biofilm Morphology of L. monocytogenes

Biofilms were observed using fluorescence microscopy according to previously published methods [22]. Sterile coverslips were positioned in 6-well plates, into which 3 mL of a bacterial solution combined with TSB-YE medium was dispensed per well. The plates were then incubated at 37 °C for 1–4 days, with each strain being repeated 3 times. TSB-YE medium without any bacterial solution was used as a blank control. Take out the slides, wash them twice with double distilled water, and air dry them at 25 °C. After air-drying, 0.1% isothiocyanic acid fluorescent staining solution (MedChemExpress, Monmouth Junction, NJ, USA) was added to each well for staining, and staining was protected from light for 15 min. Suck off the dyeing solution, wash it twice with double distilled water, and air dry it again. Finally, the coverslip was removed from the 6-well plate and the morphology of the biofilm was observed under a fluorescence microscope (Olympus, Tokyo, Japan).

### 2.4. Determination of Surface Hydrophobicity of L. monocytogenes (Microbial Hydrocarbon Adsorption Capacity Method)

The hydrophobicity of bacterial surfaces was determined according to the microbial hydrocarbon adsorption method [23]. The cultured *L. monocytogenes* solution was centrifuged at 12,000 rpm (4530R Cryogenic high-speed centrifuge, Eppendorf, Hamburg, Germany) for 2 min, washed in phosphate-buffered saline (PBS) for 2 times, resuspended, and the concentration of the bacteria solution was adjusted to be in the range of 0.8~1.0 at OD_600_ nm, which was recorded as OD_1_. Bacterial suspension and hydrophobic solvent (chloroform/xylene solution, obtained from Sinopharm Chemical Reagent Co., Ltd.) are mixed in a 5:1 ratio in test tubes and allowed to stand at 25 °C for 1 h, with PBS as the blank control, and each sample was repeated 3 times, and the OD_600_ nm was measured, which was recorded as OD_2_. The surface hydrophobicity of each strain was calculated based on the change in absorbance of the bacterial solution before and after the addition of the hydrophobic solvent, using the following equation:

Surface hydrophobicity of the strain = (1 − OD_2_/OD_1_) × 100% (1)

### 2.5. Determination of the Self-Agglutination Rate of L. monocytogenes Strains

We prepared PBS to resuspend the bacterial solution according to the method of 2.4 and adjusted the OD_600_ value of the bacterial solution to 0.8–1.0, which was recorded as OD_1_. We then added 4 mL of the adjusted concentration of the bacterial suspension to the test tube, let it stand for 1, 3, and 5 h at room temperature, respectively, and measured its OD_600_ nm by absorbing the upper solution after standing, which was recorded as OD_2_, and measurement of each sample was repeated 3 times. According to the difference in absorbance of the bacterial solution before and after standing, the self-agglutination rate of each strain was calculated, and the formula was as follows [24]:

Strain self-agglomerate = (1 − OD_2_/OD_1_) × 100% (2)

### 2.6. Determination of Protein Content in Biofilms of L. monocytogenes

We grew the biofilm according to Section 2.2, and the supernatant of the cultured biofilm was discarded, and 180 μL of double-distilled water was added to the remaining biofilm. The mixture was thoroughly mixed and this process was repeated three times for each sample. According to the instructions of the BCA protein concentration determination kit (Enhanced BCA Protein Assay Kit, Beyotime Shanghai, China [25]), the BCA working solution was configured, and the protein standard, the sample to be tested and the BCA working solution were mixed at a ratio of 1:8 and placed in a single well in a 96-well plate, incubated at 37 °C for 30 min, and the OD_562_ nm was measured. A standard curve was plotted based on the absorbance value of the protein standard. Using this standard curve, the concentration of extracellular protein in the biofilm of *L. monocytogenes* was calculated.

### 2.7. Determination of Exopolysaccharide Content in Biofilm of L. monocytogenes (Phenol-Sulfuric Acid Method)

Extraction of exopolysaccharides: the supernatant of the cultured biofilm was discarded, 200 μL of double-distilled water was added to a water bath at 100 °C for 15 min, and naturally cooled to room temperature; 85% trichloroacetic acid solution (ThermoFisher Scientific, Waltham, MA, USA) was added, and then it was allowed to stand in ice water for 30 min and centrifuged at 12,000 rpm for 20 min. We combined the supernatants and added an equal volume of absolute ethanol. The mixture was left at −20 °C for 1 h, centrifuged at 12,000 rpm for 20 min, and dissolved in 1 mL of deionized water to form an exopolysaccharide solution. We configured different concentrations of glucose solutions (10, 20, 40, 60, 80, 100, 120, 140, 160, 180, 200 μg/mL), added the collected biofilm polysaccharide samples, glucose solutions of different concentrations and double-distilled water (blank control) to 96-well plates, added 5% phenol solution (Merck, Darmstadt, Germany), added concentrated sulfuric acid, mixed well, and placed it at room temperature for 30 min; we repeated measurement of each sample 3 times, and measured OD_490_ nm. A standard curve was drawn using the absorbance value corresponding to different concentrations of glucose solution. Then, the amount of exopolysaccharides in the sample was calculated [26].

### 2.8. Determination of Extracellular DNA Content in Biofilms of L. monocytogenes

To determine the DNA content released by biofilm cells, the biofilm of the *L. monocytogenes* strain under investigation was cultured following the method outlined in Section 2.2. Subsequently, the supernatant and the lower biofilm in the well plate were collected separately. The collected supernatant was transferred to a centrifuge tube and centrifuged at 12,000 rpm for 4 min. The resulting supernatant was then combined with a protein precipitation solution composed of phenol, chloroform, and isoamyl alcohol in a ratio of 25:24:1. After centrifuging at 12,000 rpm for 10 min, the supernatant was collected [27]. Next, the collected supernatant and the prepared propidium iodide (PI) solution were mixed in a 1:1 ratio and added to the wells of a black microplate labeling plate. Each sample was prepared in five replicates for consistency and accuracy in the subsequent analysis. We placed the microplate with the sample in the dark, and left it at room temperature for 5 min. Sample fluorescence values were detected using a fluorescence microplate reader (excitation and emission wavelengths of 535 nm and 615 nm for the PI-DNA complex, respectively). The fluorescence values of the gradient standard concentration (calf thymus DNA) solution and the blank control (TE buffer) were determined simultaneously. We drew a standard curve with the fluorescence intensity corresponding to the standard and calculated the concentration of DNA released into the supernatant and biofilm after film formation by *L. monocytogenes* according to the standard curve.

### 2.9. Detection of Drug Resistance of L. monocytogenes before and after Film-Forming

The selected antibiotics are listed in Table 1 in accordance with the instructions provided for the bacterial drug minimal inhibitory concentration (MIC) test plate (Meihua International Medical Technologies Co., Ltd., Yangzhou, China). To prepare bacterial suspensions, pure cultures of *L. monocytogenes* before and after biofilm formation were used. These cultures were gently ground on the surface of the dilution flask to prepare a bacterial suspension at a concentration of 0.5 Mcfarland Standard (MCF). We took 50 μL of bacterial suspension, added it to Müller–Hinton agar (M-H) broth medium, mixed it well, and added 100 μL per well to the test plate antimicrobial susceptibility wells. The parafilm was sealed, and the accuracy of the quality control strain ATCC 19115 was evaluated according to the instructions and the standard interpretation results of the American Society for Clinical (ASCO) and Laboratory Standards after 18 h at 37 °C.

### 2.10. Effects of L. monocytogenes on Cell Adhesion and Invasion before and after Film-Forming

Human colon cancer glandular cells (Caco-2) were preserved in a −80 °C freezer or liquid nitrogen in the laboratory of the School of Life Sciences, Shanghai University. Caco-2 cells are used to mimic the tight junctions of small intestinal epithelial cells as a barrier for bacteria to invade the body [28]. Cell adhesion assay: Cells were seeded in 24-well plates, a suspension of bacteria before and after membrane formation of *L. monocytogenes* was prepared, and the bacteria were inoculated into cell culture plates for 1 h at 37 °C and 5% CO_2_. After washing three times with PBS to remove non-adherent bacteria, the cells were lysed using 0.2% Triton X-100 for 5 min. Then, samples were aspirated and subjected to serial dilution. The diluted samples were then plated onto TSA-YE plates for colony counting. Each sample was repeated 3 times, and the adhesion rate of bacterial infection cells was calculated according to the following equation:

Adhesion rate (%) = number of intracellular and extracellular bacteria/number of inoculated bacteria × 100%(3)

Cell invasion experiment [29]: bacteria were seeded in cell culture plates at 37 °C, and 5% CO_2_ co-culture infection 1 h, PBS was washed 3 times, and RPMI 1640 (ThermoFisher Scientific) medium containing 100 μg/mL gentamicin was added to a 24-well plate and incubated for 2 h to kill extracellular *L. monocytogenes*. After 3 times of PBS washing, 0.2% Triton X-100 (Beyotime) was added to lyse the cells; after 5 min of reaction, the samples were aspirated, and the gradient dilution was coated on TSA-YE plates for colony counting. Each sample was repeated 3 times, and the invasion rate of bacteria-infected cells was calculated according to the following equation:

Invasion rate (%) = number of intracellular bacteria/number of inoculated bacteria × 100%(4)

### 2.11. Detection of Gene Expression Related to Biofilm Formation of L. monocytogenes

The biofilm of the *L. monocytogenes* strain under investigation was cultured following the method described in Section 2.2. The culture vessel in the culture method was replaced with a 6-well plate, and the RNA before and after film formation of the strain was extracted according to the method of the instructions using the bacterial total RNA extraction kit (Beijing Tianmo Sci&Tech Development Co., Ltd., Beijing, China). After the RNA purity was qualified, the cDNA was reverse transcribed and set aside at −20 °C.

Biofilm-related primers were designed according to the genome sequence of *L. monocytogenes*, and 16S rRNA was used as the internal reference gene; the primer sequences are shown in Table 2. Using the reverse transcript product cDNA as a qPCR template, we added the described components using the SYBR Premix Ex Taq II kit (Takara, Kusatsu, Japan) and replicated it 3 times per sample. The required volume for each component is shown in Table 3. After mixing, it was placed in a PCR machine (CFX Real-time PCR, BIO-RAD, Hercules, CA, USA) and repeated for 35 cycles at 95 °C for 10 s, 57 °C for 15 s, and 72 °C for 60 s.

### 2.12. Statistical Analysis

Statistical analysis of the data results was conducted using one-way analysis of variance (ANOVA). All experiments were repeated a minimum of three times to ensure the reliability and consistency of the findings. Statistical analysis was carried out utilizing GraphPad 6.01 software for initial analysis, while SPSS v. 20.0 was employed for further data analysis. *p* ≤ 0.05 was considered statistically significant.

## 3. Results

### 3.1. Detection of the Biofilm-Forming Capacity of L. monocytogenes

**Figure 1:**
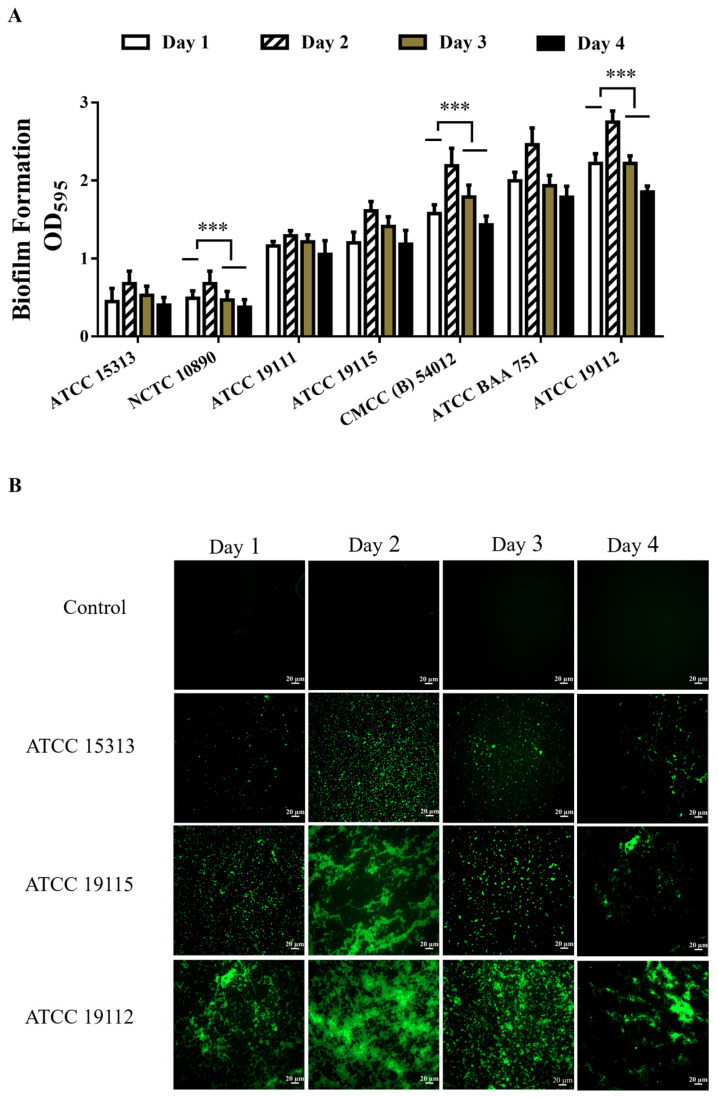
Detection of biofilm formation ability of *L. monocytogenes*. (**A**) Detection of biofilm formation ability of *L. monocytogenes*. (**B**) Observation of *L. monocytogenes* biofilm morphology by fluorescence microscopy. *** *p* < 0.001.

The biofilm-forming capacity of *L. monocytogenes* was evaluated by crystal violet staining. A higher degree of crystal violet dye binding corresponded to a greater value of OD_595_ after destaining. After 1–4 days of culturing seven standard strains of *L. monocytogenes* to assess their quantitative biofilm formation capacity, as Figure 1A illustrates, we saw that the biofilm formation of *L. monocytogenes* increased after 2 days of culture compared to that after 1 day, followed by a subsequent decline in biofilm formation capacity after the third day. Based on the film-forming ability on the second day, a hierarchy of film-forming capacity among the strains emerged: ATCC 15313 < NCTC 10890 < ATCC 19111 < ATCC 19115 < CMCC (B) 54012 < ATCC BAA 751 < ATCC 19112. Notably, strain ATCC 15313 exhibited comparatively weaker biofilm-forming ability, whereas strain ATCC 19112 strain displayed relatively robust biofilm-forming capacity.

### 3.2. Observation of Biofilm Morphology of L. monocytogenes by Fluorescence Microscopy

In order to directly observe the formation of biofilms, strains ATCC 19112, ATCC 19115, and ATCC 15313, which have strong, medium, and weak film-forming capacities, respectively, were cultured on coverslips in 6-well plates for 1–4 days. Subsequently, FITC fluorescence staining was performed, and samples were examined under a fluorescence microscope. The results are shown in Figure 1B, where the biofilm is stained green and the cells clump together to form a dense biofilm compared to the control group. The results were consistent with the crystal violet staining results, and the biofilm density was higher after 2 days of culture. ATCC 15313 forms a lower density of biofilms, and ATCC 19112 forms a higher density of biofilms.

### 3.3. Detection of L. monocytogenes Hydrophobicity and Self-Coagulation Capacity

**Figure 2:**
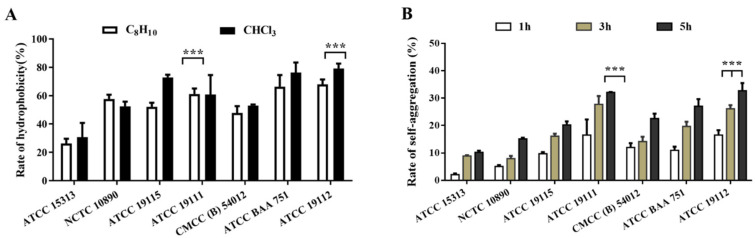
Detection of surface hydrophobicity and self-agglutination ability of *L. monocytogenes*. (**A**) Surface hydrophobicity. (**B**) Self-agglutination ability. *** *p* < 0.001.

As depicted in Figure 2A, xylene exhibited significantly lower hydrophobic compared to chloroform in different strains. Remarkably, the strain with the weakest film-forming ability had the lowest hydrophobicity and the strongest film-forming ability of the strain with higher hydrophobicity. However, no significant correlation was observed between cell surface hydrophobicity and biofilm formation. Figure 2B shows the agglutination rate measured by the strain after standing for 1, 3, and 5 h, and it can be seen that with the increase in time, the self-agglutination capacity of the seven strains showed an upward trend, and the self-agglutination rate of ATCC 19112 strain reached 32.69% at 5 h, and the self-agglutination ability of this strain was significantly higher than that of ACCC 15313 strain, with weak film-forming ability.

### 3.4. Detection of Extracellular Polymers in Biofilms of L. monocytogenes

**Figure 3:**
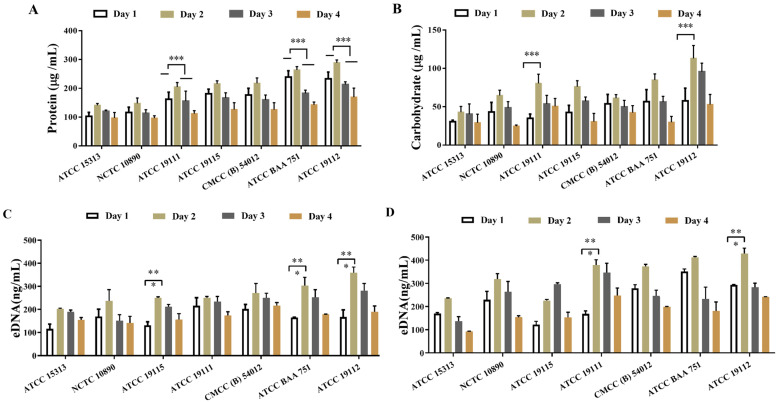
Detection of extracellular polymer content in biofilms of *L. monocytogenes*. (**A**) Extracellular protein content. (**B**) Extracellular polysaccharide content. (**C**,**D**) Extracellular DNA content. * *p* < 0.05. ** *p* < 0.01. *** *p* < 0.001.

The extracellular protein and extracellular polysaccharide contents of 7 strains of *L. monocytogenes* after film-forming were detected by BCA and phenol–sulfuric acid methods, respectively. The results, shown in Figure 3, indicate a gradual increase in both extracellular protein (Figure 3A) and exopolysaccharide (Figure 3B) contents as the biofilm matures, with a pronounced acceleration observed between 24 and 48 h during the maturation phase. At this juncture, the contents of extracellular polysaccharides and proteins in the ATCC 19112 strain were, respectively, 1.23 and 1.98 times higher than the values before film formation. Later, as the biofilm was shed and decomposed, the content of extracellular proteins and polysaccharides began to decrease. These findings underscore the pivotal role of extracellular polymer production in the maturation stage of biofilms.

Next, we detected the extracellular DNA content in the supernatant and biofilm of *L. monocytogenes* supernatant and biofilm after film formation by using PI staining, and the detection results are shown in Figure 3C,D. As can be seen in the figure, a substantial quantity of DNA was generated following biofilm formation by the strain, with DNA levels exceeding 90 ng/mL in both the supernatant (Figure 3C) and the biofilm matrix (Figure 3D). The content of extracellular DNA released into the supernatant subsequent to biofilm formation was significantly lower than that retained within the biofilm matrix, indicating that while some DNA was released from the supernatant after the formation of the biofilm, the majority remained integrated within the biofilm matrix alongside extracellular proteins and polysaccharides. Except for the ATCC 19115 strain, which exhibited peak DNA content on the 3rd day of culture, the content of extracellular DNA peaked on the 2nd day before declining, consistent with the trend of biofilm formation, indicating the significant role of extracellular DNA in the formation of biofilm in *L. monocytogenes*.

### 3.5. Effect of L. monocytogenes on Cell Adhesion Invasion before and after Film-Forming

**Figure 4:**
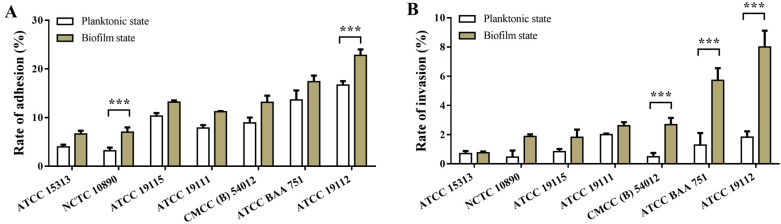
Effects of *L. monocytogenes* on cell adhesion and invasion before and after biofilm formation. (**A**) Cell adhesion. (**B**) Cell invasion. *** *p* < 0.001.

The adhesion and invasion abilities of *L. monocytogenes* to Caco-2 cells were correlated with the ability of biofilm formation, and the results are shown in Figure 4. It can be seen from the figure that the effects of *L. monocytogenes* with different film-forming abilities on cell adhesion (Figure 4A) and invasion (Figure 4B) are different, and the effect of bacteria on cell adhesion and invasion is significantly enhanced with the enhancement of film-forming ability. Compared with the planktonic state, the adhesion rate and invasion rate of the biofilm state were significantly increased, and the adhesion rate and invasion rate of ATCC 19112, the strain with the strongest film-forming ability, increased by 37% and 337%, respectively, underscoring the capacity of biofilm to augment the adhesion and invasion ability of *L. monocytogenes*, consequently amplifying its pathogenicity.

### 3.6. Detection of Drug Resistance of L. monocytogenes before and after Film-Forming

Fourteen antibiotics were selected to evaluate the resistance of *L. monocytogenes* before and after film formation. It can be seen from Table 4 that 7 strains of *L. monocytogenes* showed sensitivity to ampicillin, penicillin, oxacillin, cotrimoxazole, vancomycin, gentamicin, and imipenem. With the exception of ATCC 15313, all strains demonstrated resistance to cefoxitin, whether in planktonic or film-forming states. Despite an increase in the MIC value of *L. monocytogenes* following biofilm formation compared to their pre-biofilm state, they were still susceptible to ampicillin, penicillin, oxacillin, cotrimoxazole, vancomycin, and gentamicin. The drug resistance of the film-forming strains was enhanced, and erythromycin, clindamycin, ciprofloxacin, and daptomycin all changed from sensitive to moderately sensitive and drug-resistant, and the film-forming strains all developed resistance to cefoxitin.

### 3.7. Detection of Gene Expression Related to Biofilm Formation of L. monocytogenes

**Figure 5:**
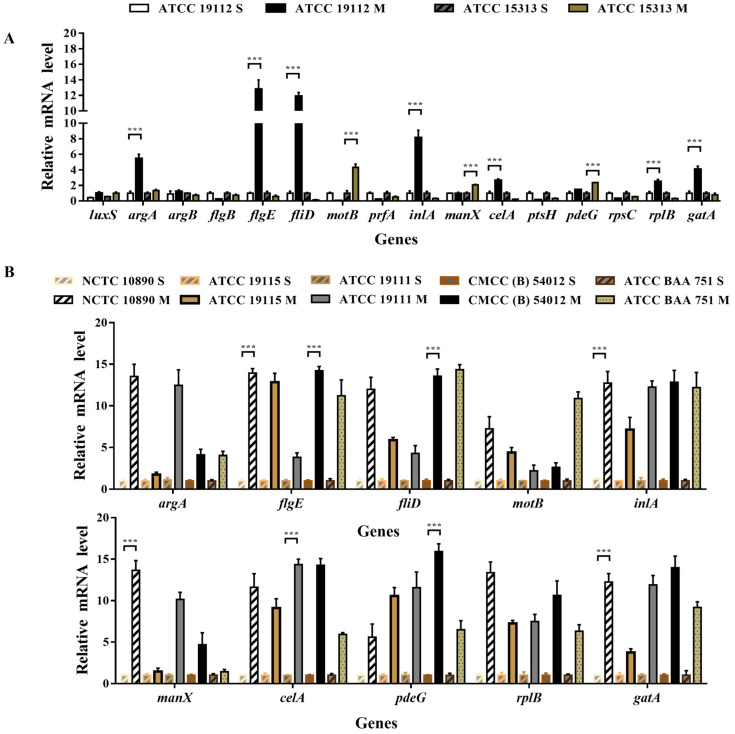
Expression of genes related to *L. monocytogenes* biofilm formation. S indicates the pre-film-forming state of the strain, and M indicates the post-film-forming state of the strain. (**A**) QRT-PCR was used to detect the expression of biofilm-related genes in the planktonic state and biofilm of the strain with strong film-forming ability (ATCC 19112) and the weak strain (ATCC 15313). (**B**) QRT-PCR was used to detect the gene expression levels of 10 genes with large differences before and after film formation in different strains of plankton and biofilm. *** *p* < 0.001.

RT-PCR was used to detect the expression of related genes before and after biofilm formation in the strong film-forming strain (ATCC 19119) and the weakly film-forming strain (ATCC 15313). It can be seen from Figure 5A that significant divergent gene expression patterns were observed among strains with varying film-forming abilities. ATCC 19112 was significantly up-regulated by genes in the QS system (*argA*), flagella (*flgE*, *filD*), virulence (*inlA*), and extracellular polymer (*pdeG*), especially by flagella-related genes. Conversely, ATCC 15313 gene was not significantly down-regulated in the strain with weak film-forming ability, and was regulated by flagella-related genes (*motB*) and extracellular polymer (*manX*)-related genes.

On this basis, we selected 10 genes with significant expression differences and used RT-PCR to detect the rest of the strains. Figure 5B illustrates the results, with consistent and significant upregulation of all tested genes after membrane formation, emphasizing their important role in the biofilm formation process.

## 4. Discussion

Bacteria employ extracellular polymer secretion to encapsulate themselves within biofilms, a complex and dynamic process regulated by a variety of factors. Biofilm formation involves a variety of changes in bacterial motility, adhesion, extracellular polymer secretion, and energy conversion.

The auto-aggregation and surface hydrophobicity of bacteria are some of the most important physicochemical properties of microbial cells, playing a significant role in both the invasion of intestinal epithelial cells and the non-specific adhesion to various surfaces. Prior research has indicated that strains with increased hydrophobicity exhibit enhanced adherence to polystyrene surfaces [30]. Cell aggregation within the same strain, which involves mutual recognition and self-aggregation, facilitates the formation of microcolonies on host cell surfaces. The mutual recognition and adhesion between cells also exert a profound influence on biofilm formation [31]. Those findings are to some extent consistent with the results of this study, as the strains with the weakest biofilm formation also have poor hydrophobicity and self-aggregation ability (Figure 2). According to our findings, hydrophobicity facilitates adhesion, which is a crucial step in the initial formation of biofilms. However, it may not have a strong correlation with the subsequent growth and maturation of biofilms. Extracellular polymers, primarily composed of polysaccharides, proteins, and DNA, serve as essential scaffolds for biofilms, contributing to the formation of their structures [32]. Research by Sadovskaya et al. has highlighted teichoic acid as a primary component of exopolysaccharides, which can be absorbed into biofilm extracellular polysaccharides when bacteria adhere to contact surfaces [33]. This process plays a significant role in the formation and structure of biofilms, impacting their adhesion and stability on various surfaces. Volkan et al. demonstrated that the second messenger, c-di-GMP, could promote the synthesis of exopolysaccharides from *L. monocytogenes*, thereby promoting biofilm formation [34]. This is similar to the results of this study. We discovered significant differences in the content of exopolysaccharides among different *L. monocytogenes* strains with different biofilm-forming ability, and the more biofilm-forming strains have more exopolysaccharide content. (Figure 3A).

Extracellular proteins are also one of the important components of bacterial biofilms. Treating *L. monocytogenes* biofilms with proteolytic enzymes completely inhibits or significantly reduces the formation of biofilms, making it almost lose the matrix structure [35]. The extracellular matrix protein CdrA of *Pseudomonas aeruginosa* has been shown to bind to extracellular polysaccharides, promoting aggregate stability and thus accelerating biofilm formation [36,37]. In this study, we found that the content of extracellular proteins correlated with biofilm formation ability and with the content of extracellular polysaccharides. We hypothesized that extracellular proteins of *L. monocytogenes* also play a role in biofilm formation in conjunction with extracellular polysaccharides.

Furthermore, research by Morten et al. [38] showed that extracellular DNA binds very tightly to bacteria and is essential for the formation of biofilms in *L. monocytogenes*. The addition of DNase I significantly reducing cell attachment, resulting in reduced biofilm formation. DNA was also found in the supernatant of *L. monocytogenes* during biofilm culture. It is generally believed that such extracellular DNA is produced by both active secretion and cell lysis in *L. monocytogenes* and other bacteria [27,39]. In this study, the content of extracellular DNA in the biofilm and supernatant of *L. monocytogenes* was also investigated. We found that the content of extracellular DNA in the biofilm was greater than that in the supernatant. The extracellular DNA content of the strain with strong film-forming ability was much higher than that of the strain with weak film-forming ability (ATCC 15313). The content was the highest at 2 days of culture, which was consistent with biofilm formation. We posit that the level of extracellular DNA affects biofilm formation in *L. monocytogenes*, and DNA released into the supernatant may facilitate efficient attachment to surfaces.

*L. monocytogenes* is a foodborne pathogen that usually enters the host through ingestion of contaminated food and infects the intestine. Therefore, the invasion of intestinal epithelial cells is a critical step in exerting its pathogenic role. Several researchers have utilized various cell lines such as Caco-2, Vero, and HT-29 to investigate the adhesion and invasion ability of *L. monocytogenes* [40,41]. Caco-2 cells, resembling human intestinal epithelial cells morphologically, are commonly employed for such studies. In our investigation, we examined whether seven strains of *L. monocytogenes* with different biofilm-forming abilities affect the pathogenicity of bacteria, and found that the strains with strong biofilm-forming ability had significantly higher adhesion and invasion ability to cells in vitro. This finding is consistent with previous research that strains with strong adhesion may be more invasive than strains with weak adhesion [42].

The primary antibiotics currently employed clinically for treating *L. monocytogenes* infection are penicillin, ampicillin, or in combination with gentamicin to produce synergistic effects [43]. Initially, *L. monocytogenes* was susceptible to most antibiotics in the early stages, but it was found that *L. monocytogenes* gradually became resistant to one or more antibiotics [44,45], especially when *L. monocytogenes* forms biofilms as biofilms enhance the resistance of associated cells to antibiotic drugs. Chen et al. [2] conducted antimicrobial susceptibility tests on 362 strains of *L. monocytogenes* isolated from meat products, among which the resistance rates to ampicillin and tetracycline were 40.0% and 11.8%, respectively. The intermediate sensitivity rate of ciprofloxacin was 45.0%, and the drug resistance rate was 4.6%. In this study, antimicrobial susceptibility experiments on *L. monocytogenes* showed that all seven strains were susceptible to ampicillin, penicillin, oxacillin, cotrimoxazole, vancomycin, gentamicin and imipenem, and all of them were resistant to cefoxitin except ATCC 15313. The antibiotic resistance of most plankton bacteria is also applicable to individual cells within biofilms, and film formation significantly increases drug resistance. *L. monocytogenes* gains increased resistance to benzalkonium chloride, peracetic acid, and lactate during the formation of mature biofilms [46]. Our data show that the MIC value of the strain increases after the formation of the biofilm, and the antimicrobial resistance of the strain is also enhanced. Specifically, strains transitioned from susceptible to moderately sensitive or resistant to erythromycin, clindamycin, ciprofloxacin, and daptomycin. Furthermore, all biofilm-forming strains developed resistance to cefoxitin. Therefore, the relationship between biofilm formation capacity and antimicrobial resistance is controversial and needs to be further studied.

In this study, the expression levels of genes associated with biofilm formation across strains with varying film-forming abilities were investigated both before and after biofilm formation. The related genes such as QS system, flagella, virulence, and extracellular polymers that may affect the biofilm formation of *Listeria monospurum* were selected to explore their roles in biofilm formation. Aurélie et al. [47] showed that the bacterial adhesion capacity of agrA and agrD knockout strains was reduced, and their biofilm production was significantly reduced in the 24 h prior to incubation on polystyrene. Consistent with this, we found that the expression level of *agrA* gene in the strain with strong biofilm-forming capacity (ATCC 19112) was up-regulated by 5.4-fold after biofilm formation, but there was no differential expression in the strain with poor film-forming ability. This finding fully demonstrated that *agrA* gene plays a positive regulatory role in biofilm formation process. Zhang et al. found that the amount of biofilm formation was reduced in the *luxS* gene deletion strain of *L. monocytogenes*, but the *luxS* gene expression did not differ between the two strains in this study. This may be caused by the differences in biofilm formation capacity due to differences in strains [48]. Berlage et al., in their study of *Vibrio parahaemolyticus*, found that the absence of *flgD* and *flgE* flagellar genes hindered the formation of mature biofilms [49]. *FlgJ* is a glycoside hydrolase (GH) that belongs to the carbohydrate-active enzyme family and plays a very important role in flagellar assembly; *flhE* is present in some Cronobacter (about 34.8%), which promotes biofilm formation [50]. In this study, the flagellar formation-related genes *flgE* and *fliD* were up-regulated by more than ten times in strains with strong film-forming. Conversely, the *motB* gene was significantly up-regulated in the strains with weak film-forming. Those findings together suggest that flgE and *fliD* could promote biofilm formation, while *motB* negatively regulated biofilm formation. Furthermore, the eight-fold up-regulation of the *L. monocytogenes* virulence factor *inlA* in the film-forming strain suggests a strong correlation between biofilm formation and pathogenicity [51]. In addition, the genes related to the formation of extracellular polymers were also detected, and the expression of these genes showed varying degrees of upward and downward regulation. Admittedly, the biofilm formation process was intricate and regulated by multiple gene pathways, and qPCR could not fully analyze them.

## 5. Conclusions

In this study, we found that the biofilm-forming capacity of *L. monocytogenes* was strongest at two days of culture. At the same time, we found that the strains with stronger membrane ability had stronger self-agglutination ability and more vigorous extracellular polymer secretion, while there was no significant correlation between surface hydrophobicity and biofilm formation. In addition, we found that the adhesion and invasion of cells and the increase of drug resistance after biofilm formation increased, and we speculated that the formation of biofilm makes *L. monocytogenes* more virulent and more difficult to be removed by biocides, which poses a huge safety hazard to food safety. Moreover, our investigation revealed elevated expression levels of genes associated with biofilm formation post film development, including those involved in quorum sensing, flagellar synthesis, and extracellular polymer synthesis. These findings shed light on the mechanisms underpinning the increased virulence and antimicrobial resistance of *L. monocytogenes* biofilms, emphasizing the critical importance of effective biofilm control strategies in ensuring food safety.

## Figures and Tables

**Table 1:** Class and name of antibiotics.

Drugs	Abbreviation	Drugs	Abbreviation
Ampicillin	AMP	Sulfamethoxazole/Trimethoprim	T/SUL(SXT)
Penicillin	PEN	Vancomycin	VAN
Oxacillin	OXA	Tetracycline	TET
Erythromycin	ERY	Chloramphenicol	CHL
Clindamycin	CLI	Gentamicin	GEN
Ciprofloxacin	CIP	Cefoxitin Mefoxin	FOX(CFX)
Daptomycin	DAP	Imipenem	IPM

**Table 2:** qRT-PCR primer sequence.

Primer	Sequence (5′-3′)	Primer	Sequence (5′-3′)
16sRNA-F	AAAGAGAGTTTGATCCTGGCTC AGGACG	*inlA*-F	CGGTGGAATTCAGTATTCAAACTAGTTTTAATGG
16sRNA-R	AAAGGAGGTGATCCAGCCGCAC	*inlA*-R	CTTTGATTGTTTTGCGGAGAATT AGCGTC
*luxS*-F	GGCAGAAAAAATGAATGTAGAAAGTTTTAATTTAGACCAT	*manX*-F	GGTAGGAATTATCCTCGCAACTCACGG
*luxS-R*	CACCAAACACATTTTTCCATT CGCTTCG	*manX*-R	GATGATATCTTCCATGTTAGCGC TTGAGTCA
*argA*-F	GTGAAGATAACAGAATGCAG CGAGAAAGG	*celA*-F	TCATGTTAGTATGTTCAGCAGGT ATGTCTACC
*argA*-R	CAAGCTTTTAATTAATTTCGATGATGCATAACAATTTTCAC	*celA*-R	CATTAACTCTAATGCTTGTTCTAAAACTTTGTCGCC
*argB*-F	GTCCCTTTGTCAGAAAGAATGGCG	*ptsH*-F	ATGGAACAAGCAAGTTTTGTAGT AATCGATGA
*argB*-R	CATAGTTCCGATACCTCCTTTTCAATAGTTTGT	*ptsH*-R	CAGCCAATCCTTCTTTCTTAAGAACTTCAGTTAG
*flgB*-F	GTGGAAAATTACACCACGCATAT TGGC	*pdeG*-F	ATGAAAAAGCCCTCCGTACGTGA GATTATT
*flgB*-R	TTACTTTCCACGGGCTGCTGTGT	*pdeG*-R	CCTTGCGCATAGGGAATGCCGATTT
*flgE*-F	CTATGTATACAGCTATTTCTGGG ATGAATGCG	*rpsC*-F	GTACATCCAATAGGTATGCGTATCGGTG
*flgE*-R	GTTCACAATTTGTTTCATCACGT CATCCGC	*rpsC*-R	CCTCCTTCCACATTGTTTTTCT TCGTAGG
*fliD*-F	GTCAAGAACAAATTGACGCCCTGCT	*rplB*-F	GTATAAACCTACCACTAACGGGCGCC
*fliD*-R	TAGAGCGGCGGCGTAACGTAC	*rplB*-R	ACGACGACGTACGATAAATTT ATCGGAG
*motB*-F	GTGGCCAAGCGTCGCAAGAA	*gatA*-F	CGTAGTTTGTACAGTCTTCAGTA TCATCGG
*motB*-R	CTACTCATCTTCATCAAGCG TATCGCG	*gatA*-R	CTATTTTGCGATTGTTGCTCATAGGCAT
*prfA*-F	ATGAACGCTCAAGCAGAAGAATT CAAAAAAT		
*prfA*-R	CCCCAAGTAGCAGGACATGCTAAAT		

**Table 3:** qPCR reaction system.

System Components	Volume/(μL)
cDNA template	1
SYBR Premix	12.5
Upstream and downstream primers (10 μM)	2
DEPC	9.5

**Table 4:** Resistance test results of *L. monocytogenes* before and after biofilm formation.

Antibiotic	Status	MIC (μg/mL)/Sensitivity						
		ATCC 15313	ATCC 10890	ATCC 19111	ATCC 19115	CMCC (B) 54012	ATCC BAA 751	ATCC 19112
**AMP**	S	<0.125/S	0.125/S	<0.125/S	0.25/S	0.25/S	0.25/S	1/S
	M	2/S	0.5/S	1/S	0.5/S	0.5/S	0.25/S	1/S
**PEN**	S	0.0625/S	0.125/S	0.0625/S	0.5/S	0.5/S	0.25/S	0.5/S
	M	0.25/S	0.125/S	0.25/S	1/S	2/S	0.25/S	0.5/S
**OXA**	S	<0.25/S	2/S	0.5/S	2/S	2/S	2/S	1/S
	M	2/S	2/S	0.5/S	2/S	2/S	2/S	2/S
**ERY**	S	0.5/S	0.25/S	0.5/S	1/I	2/I	1/I	4/I
	M	1/**I**	2/**I**	1/**I**	2/I	4/I	8/**R**	8/**R**
**CLI**	S	0.25/S	0.25/S	1/I	0.5/S	2/I	4/R	2/I
	M	1/**I**	0.5/S	2/I	2/**I**	2/I	4/R	2/I
**CIP**	S	0.5/S	0.5/S	2/I	4/R	1/S	1/S	4/R
	M	2/**I**	1/S	2/I	4/R	4/**R**	2/**I**	4/R
**DAP**	S	0.125/S	2/R	1/S	1/S	1/S	2/R	1/S
	M	1/S	2/R	1/S	2/**R**	2/**R**	4/R	4/**R**
**T/SUL(SXT)**	S	(0.25/ 4.75)/S	(0.25/ 4.75)/S	(0.25/ 4.75)/S	(0.25/ 4.75)/S	(0.25/ 4.75)/S	(0.25/ 4.75)/S	(0.25/ 4.75)/S
	M	(0.25/ 4.75)/S	(0.25/ 4.75)/S	(0.25/ 4.75)/S	(0.25/ 4.75)/S	(0.25/ 4.75)/S	(0.25/ 4.75)/S	(0.25 /4.75)/S
**VAN**	S	<0.5/S	0.5/S	0.5/S	0.5/S	0.5/S	0.5/S	0.5/S
	M	1/S	0.5/S	2/S	1/S	1/S	1/S	2/S
**TET**	S	8/I	4/S	16/R	8/I	8/I	16/R	8/I
	M	8/I	8/**I**	16/R	8/I	8/I	16/R	16/**R**
**CHL**	S	16/I	8/S	4/S	4/S	16/I	8/S	16/I
	M	32/**R**	8/S	4/S	16/**I**	16/I	16/**I**	32/**R**
**GEN**	S	<1/S	1/S	<1/S	<1/S	<1/S	<1/S	<1/S
	M	4/S	1/S	2/S	1/S	4/S	1/S	1/S
**FOX(CFX)**	S	4/S	>8/R	>8/R	>8/R	>8/R	>8/R	>8/R
	M	>8/**R**	>8/R	>8/R	>8/R	>8/R	>8/R	>8/R
**IPM**	S	<1/S	<1/S	<1/S	<1/S	<1/S	2/S	<1/S
	M	1/S	1/S	1/S	2/S	1/S	8/**I**	1/S

Note: S represents the pre-film-forming state of *L. monocytogenes*, and M represents the post-film-forming state of *L. monocytogenes*. R indicates resistance (orange), I indicates moderate sensitivity (light orange), and S indicates sensitivity (white). Bold red letters indicate that resistance changes from sensitive to moderately sensitive, resistant, or moderately sensitive to resistant.

## Data Availability

The original contributions presented in the study are included in the article, further inquiries can be directed to the corresponding authors.
